# Investigating the effect of child maltreatment on early adolescent peer-on-peer sexual aggression: testing a multiple mediator model in a non-incarcerated sample of Danish adolescents

**DOI:** 10.3402/ejpt.v5.24533

**Published:** 2014-06-26

**Authors:** Rikke Holm Bramsen, Mathias Lasgaard, Mary P. Koss, Ask Elklit, Jytte Banner

**Affiliations:** 1 Department of Psychology, University of Southern Denmark, Odense, Denmark; 2 Public Health and Quality Improvement, Central Region Denmark, Denmark; 3 College of Public Health, University of Arizona Tucson, AZ, USA; 4 Department of Forensic Medicine, University of Copenhagen, Copenhagen, Denmark

**Keywords:** Sexual aggression, child maltreatment, adolescence, mediator model

## Abstract

**Objective:**

The aim of the present study was to investigate the relationship between child maltreatment and severe early adolescent peer-on-peer sexual aggression, using a multiple mediator model.

**Methods:**

The study comprised 330 male Grade 9 students with a mean age of 14.9 years (*SD=*0.5).

**Results:**

Estimates from the mediation model indicated significant indirect effects of child physical abuse on sexual aggression via peer influence and insecure-hostile masculinity. No significant total effect of child sexual abuse and child neglect on sexual aggression was found.

**Conclusions:**

Findings of the present study identify risk factors that are potentially changeable and therefore of value in informing the design of prevention programs aiming at early adolescent peer-on-peer sexual aggression in at-risk youth.

Adolescent peer-on-peer sexual aggression (APSA) is being recognized as a prevalent and detrimental health problem (Foshee, Benefield, Ennett, Bauman, & Suchindran, 2004; Jackson, Cram, & Seymour, 2000; White, Kadlec, & Sechrist, 2008), with self-reported prevalence rates between 2.2 and 34% in non-criminal samples of youth (Ageton, 1983; Lodico, Gruber, & DiClemente, 1996; Maxwell, Robinson, & Post, 2003). Studies have suggested that APSA is rooted in experiences of child maltreatment (Lodico et al., 1996; Seto & Lalumière, 2010; White et al., 2008; White & Smith, 2004). However, the vast majority of maltreated children do not exhibit later sexually aggressive behaviors, which has led to a growing interest in factors that may serve as mediating variables between episodes of child abuse and later sexual assault perpetration (Casey, Beadnell, & Lindhorst, 2009; Thomas & Fremouw, 2009). Accordingly, the present study set out to test a multiple mediator model of the effect of child maltreatment, encompassing child sexual abuse (CSA), child physical abuse (CPA), and child neglect (CN) on self-reported severe APSA in a non-incarcerated sample of early adolescent Danish boys.

## Adolescent peer-on-peer sexual aggression

Most studies on the etiology of male sexual perpetration include adult offenders or samples of juvenile offenders held in custody (Seto & Lalumière, 2010). Less attention has been paid to antecedents of severe sexual aggression in non-criminal samples of adolescents (Maxwell et al., 2003; Young, King, Abbey, & Boyd, 2009). However, the study of young offenders may contribute to the understanding of precursors of sexual aggression, as a significant minority of sexual assaults is committed by juveniles. For example, according to Barbaree and Marshall (2008) approximately 20% of all rapes are committed by adolescent males.

In addition, literature has revealed different typologies between those who molest children or sexually assault peers (Boyd, Hagan, & Cho, 2000; Gunby & Woodhams, 2010; White et al., 2008). In order to develop targeted treatment and prevention approaches, researchers call for a more rigorous separation of unique subtypes according to sexual victim age, when proposing explanatory models and theories relative to adolescent perpetrators of sexual violence (Gunby & Woodhams, 2010; Seto & Lalumière, 2010).

## Child maltreatment

A history of child maltreatment has emerged as a strong and consistent risk factor of APSA across different sampling methodologies, including representative population-based surveys, school-based surveys, clinical samples, and among incarcerated juvenile sex offenders (Seto & Lalumière, 2010; White & Smith, 2004). For instance, the *sexually abused sexual abuser* hypothesis suggests that victims of CSA are at increased risk to perpetrate sexually (Seto & Lalumière, 2010). Investigating CSA as a predictor of adolescent sexual aggression, Lodico et al. (1996) found that male victims of sexual abuse were twice as likely to force sexual contact on a friend or date compared to non-abused peers. Likewise, Seto et al. (2010) established a robust link between having been sexually coerced and engaging in coercive sexual behavior, using a large sample of Swedish and Norwegian high school students. In addition, Widom and Ames (1994) found that child maltreatment, and in particular physical abuse, was predictive of subsequent arrest for sexual assault.

In a recent meta-analysis, comparing male adolescent sex offenders with male adolescent non-sex offenders, Seto and Lalumière (2010) documented that adolescent sex offenders had experienced previous sexual coercion five times more often than non-sexually offending adolescents, whereas the difference between the two groups in relation to experiences of childhood physical abuse or neglect was markedly smaller. The meta-analysis specifically compared different types of childhood maltreatment across two groups of adolescent offenders. However, according to the work of Finkelhor and colleagues (Finkelhor, Ormrod, & Turner, 2007; Finkelhor, Ormrod, Turner, & Hamby, 2005), it is common for children and youth to have experienced multiple types of childhood victimization, referred to as poly-victimization. As such, there is a risk for potential, however often undetected, cumulative and interactive effects among different kinds of childhood maltreatment (Finkelhor et al., 2007).

## Mediating variables

Although child maltreatment is linked with later sexual aggression, the vast majority of previously maltreated children do not become perpetrators of APSA (White & Smith, 2004). As such, sexual aggression may be shown to be dependent on a number of intermediate variables, rather than directly on prior experience of child maltreatment. To date, the path leading from child maltreatment to APSA in non-criminal adolescent samples is understudied. However, in young adult samples, a number of complex etiology models have suggested a mediated path from child maltreatment to later sexual aggression (Johnson & Knight, 2000; Knight & Sims-Knight, 2004, 2009; Malamuth, Linz, Heavey, Barnes, & Acker, 1995; Malamuth, Sockloskie, Koss, & Tanaka, 1991; Vega & Malamuth, 2007). Though framed to explain adult sexual aggression, such theories may provide a useful structure for studying APSA, because they are implicitly developmental in some parts (i.e., the effect of child abuse and neglect on later APSA).

Malamuth and colleagues (Malamuth et al., 1991, 1995; Vega & Malamuth, 2007) introduced the Confluence Model, which is the most widely replicated etiology model of male sexual aggression. The model examines pathways from experiences of child maltreatment (a factor that combines witnessing domestic violence and experiencing physical and/or sexual abuse) to adult sexual aggression. The model predicts sexual aggression on the basis of integrating a number of known risk factors of adult sexual aggression, organized within two separate paths: a Hostile Masculinity path, and an Impersonal Sex path. According to the Confluence Model, experiences of child maltreatment increase the risk of involvement with delinquent peer groups and activities, which reinforce subsequent negative attitudes towards women and sexual behavior. Although the model may provide a guide for conceptualizing pathways in adolescents, the unique contribution of separate elements of the Confluence Model, that is, impersonal sex, insecure-hostile masculinity, and controlling-hostile masculinity, has not been investigated in relation to APSA in an adolescent non-criminal population.

In addition, other factors have likewise been suggested as possible explanations of the path from child maltreatment to APSA. Researchers argue that early childhood experiences of abuse and neglect may affect normal developmental processes, influencing adolescent behaviors and relationships with peers and intimates (Burton, Miller, & Shill, 2002; Wolfe, Wekerle, Scott, Straatman, & Grasley, 2004). Studies have indicated that male victims of child maltreatment display more promiscuous behaviors (i.e., early sexual onset and larger number of sexual partners) than do non-victims (Black et al., 2009; Raj, Silverman, & Amaro, 2000). Also, attitudes that justify sexual violence are likely to originate from the family context, and as adolescents shift their attention toward peer groups, family attitudes may possibly influence peer relationships (Wolfe et al., 2004). Consequently, peer and intimate relationships of maltreated youth have often been characterized as mistrustful and hostile, with distorted beliefs about what constitutes healthy relationships (Wolfe, Jaffe, & Crooks, 2006).

Following such line of thinking, child maltreatment possibly sets off various developmental trajectories, which in turn predict increased risk of APSA. Accordingly, adolescent promiscuous behaviors are found to increase the risk of male sexual assault perpetration (Maxwell et al., 2003; Young, King, Abbey, & Boyd, 2009). Also, being a part of a social group that condones hostile and sexist norms can provide encouragement and justification for sexual assault, and a number of studies have indicated that male adolescents who report rape-supportive attitudes are more likely to commit forced sex against peers (Byers & Eno, 1991; de Bruijn, Burrie, & van Wel, 2006; Kershner, 1996; Lacasse & Mendelson, 2007; Lanier, 2001).

## Purpose

The present study set out to expand existing literature on APSA by addressing several of the gaps and limitations. The study aimed to test the hypothesis of a mediated relationship between three different forms of child maltreatment (i.e., sexual abuse, physical abuse, and neglect) and severe APSA among non-criminal early adolescent boys. Mediators were selected to reflect the current literature on precursors of male sexual aggression that may be sensitive to the impact of child maltreatment, and included separate elements of early sexual onset, number of sexual partners, impersonal sex, peer influence, insecure-hostile masculinity, controlling-hostile masculinity, and rape attitudes.

## Method

### Participants and procedure

Data for this study were part of The Danish Study on Adolescent Rape Prevention (DSARP; Wave 1). The sample comprised 330 male Grade 9 students (*M* age=14.9 [*SD=*0.55]) from 35 different schools, situated in the middle region of Denmark. Study questionnaires were completed during regular school hours, and were administered according to standardized instructions. Respondents were informed about the objective of the study, voluntariness of participation, and the confidentiality of their responses. Schoolteachers provided informed consent for all students, and the study protocol was approved by the school headmasters and Aarhus University (Bramsen et al., 2013).

### Measures

#### Child maltreatment

Retrospective reports on child maltreatment were obtained through the CSA, CPA, and CN items from the National Comorbidity Survey (Kessler, Sonnega, Bromet, Hughes, & Nelson, 1995). The CSA item stated: “Have you ever experienced childhood sexual abuse?”, the CPA item stated: “Have you ever experienced childhood physical abuse?”, and the CN item stated: “Have you ever experienced child neglect?”, respectively. All items were rated on a two-point (yes/no) format. The questions were based on a format previously used in a Danish youth sample (Elklit, 2002b).

#### Adolescent peer-on-peer sexual aggression

Respondents’ perpetration of severe APSA was identified through the male version of the Sexual Experience Survey (SES; Koss & Oros, 1982). The SES collects information on perpetration of unwanted sexual activity, based on behaviorally specific descriptions of acts and tactics. The original SES scale comprises 12 items rated on a two-point scale (yes/no), which was the same response format used in the present study. To reflect the extant literature (Abbey, McAuslan, Zawacki, Clinton, & Buck, 2001; Koss et al., 2007), one item was added to the SES to capture perpetration of sexual aggression when ability to consent was impaired by drugs or alcohol. The added item was phrased: “Have you ever had sex with a girl, who was so drunk or stoned, that she couldn't put up resistance” (item 13). Based on selected items, data were used to categorize respondents into two groups reflecting (1) no perpetration of severe APSA, and (2) perpetration of severe APSA. Severe APSA included perpetration of unwanted sexual intercourse involving force, threat of force, or when ability to consent was impaired by drugs or alcohol (SES; items 10–13). Severe APSA followed the Danish legal term of “rape” (Bramsen, Elklit, & Nielsen, 2009). Other types of sexual perpetration (e.g., unwanted kissing or petting, attempted sexual aggression, sexual perpetration subsequent to verbal pressure) were not included in the present analysis. Finally, because the SES was used to establish a dichotomous status variable, internal consistency was not calculated.

#### Mediators

Seven potential mediating variables were assessed: early sexual onset, number of sexual partners, impersonal sex, peer influence, insecure-hostile masculinity, controlling-hostile masculinity, and rape attitudes. Early sexual onset was assessed by asking if respondents had experienced consensual penetrative sex before the age of 14. Answers were rated in a two-point (yes/no) format. Number of sexual partners was measured by asking participants to state their total lifetime number of consensual sexual partners. In accordance with Vega and Malamuth (2007) the measurement of impersonal sex consisted of two items answered on a seven-point rating scale, ranging from *never* (1) to *every day* (7). The questions were: “How often do you become sexually stimulated, when you see an attractive girl whom you do not know?” and “How often do you masturbate?” Higher scores reflect more adherences to impersonal sex. Although the measure consists of only two items showing low internal reliability (*α=*0.52), the impersonal sex measure was included in the study because of a previously shown predictive power together with a measure of hostile masculinity (Malamuth et al., 1995). Peer influence was assessed by including a measure on Informal Support (DeKeseredy & Kelly, 1995) reflecting peer norms and attitudes that justify sexual and physical violence against a female partner (sample item: “Did any of your male friends tell you that it is alright for a man to hit his date or girlfriend in certain situations?”). The Informal Support measure consisted of seven items scored in a two-point (yes/no) format, and showed good internal consistency (*α=*0.83). Insecure-Hostile Masculinity and Controlling-Hostile Masculinity were assessed by the Hostile Masculinity Scale Brief (N. Malamuth, personal communication, March 2007). The scale consisted of 34 items answered on a seven-point Likert scale ranging from *disagree strongly* (1) to *agree strongly* (7). The Insecure-Hostile Masculinity subscale (15 items) taps into an insecure, defensive, hypersensitive, and hostile orientation, particularly toward females (sample item: “My own sexual behavior is important because I like the feeling of having another person submit to me”). The Controlling-Hostile Masculinity subscale (19 items) measures gratification from controlling females (sample item: “Being roughed up is sexually stimulating to many women”). Both subscales showed good internal consistency (Insecure-Hostile Masculinity *α=*0.76; Controlling-Hostile Masculinity *α=*80), and a moderate intercorrelation (*r*=0.42). Adherence to rape attitudes was assessed using The Attitudes toward Rape Victims Scale (Ward, 1988). This scale comprises 25 items rated on a five-point Likert scale ranging from *disagree strongly* (1) to *agree strongly* (5), with higher scores reflecting more negative rape attitudes (sample item: “Women often claim rape to protect their reputations”). The measure showed good internal consistency (*α=*75), and previous studies have provided evidence that the scale is applicable cross-culturally (Xenos & Smith, 2001), including in a Danish sample (Elklit, 2002a).

Prior to the present study all measures were adapted to Danish using a translation-back-translation procedure. Also, the questionnaire was subsequently piloted on a group of age matching students. All ambiguous items, in relation to both comprehensibility and content were discussed, which led to minor revisions of the Danish scale.

## Results

### Data analyses

The analysis estimated the total effect of CSA, CPA, and CN on APSA and the indirect effects that were mediated by variables representing early sexual onset, number of sexual partners, impersonal sex, peer influence, insecure-hostile masculinity, controlling-hostile masculinity, and rape attitudes. Prior to data analysis, the data were screened for errors. The percentage of missing values was small (0.0–5.5%). Thus, the Expectation Maximization algorithm, which has been demonstrated to be an effective method of dealing with missing data (Bunting, Adamson, & Mulhall, 2002), was used to impute missing data.

The model was tested using the approach proposed by Preacher and Hayes (2008) that allows multiple mediators to be included in the analysis. The model was specified and estimated using Mplus 5.21 (Muthén & Muthén, 1998–2009) based on maximum likelihood estimation and 1,000 bootstrap draws. Maximum likelihood estimation provides estimates that are not biased under conditions of non-normality, but the associate test statistics may be incorrect (Bollen, 1989). Therefore, the statistical significance of the mediated effects was calculated using bootstrapped bias-corrected and accelerated percentile based confidence intervals (Efron, 1987; Efron & Tibshirani, 1993). The empirically based confidence intervals used in this study should avoid making incorrect inferences about statistical significance.

The means and standard deviations of the variables are shown in Table 1. The self-report based prevalence of CSA was 1.9%, whereas 8.2 and 7.9% of the students reported the experience of CPA and CN, respectively. In addition, the reported prevalence of severe APSA was 3.6%. Whereas none from the CSA group reported severe APSA, 16.7% of those having experienced CPA and 4.2% from the CN group, also reported severe APSA. A total of 6.7% reported early sexual onset and the percentages for sex partners were as follows: Fifty-eight percent reported zero sex partners, 21% reported one, and 21% reported two or more (range 0–7).

**Table 1:** Means and standard deviations for the variables in the mediated model of childhood physical abuse and adolescent peer-on-peer sexual aggression

Variable	Mean *(SD)*
Childhood physical abuse	0.08 (0.28)
Adolescent peer-on-peer sexual aggression	0.04 (0.19)
Early sexual onset	0.07 (0.25)
Number of sexual partners	0.97 (1.60)
Impersonal sex	8.37 (3.13)
Peer influence	0.31 (0.94)
Insecure-hostile masculinity	28.39 (7.51)
Controlling-hostile masculinity	54.15 (13.55)
Rape attitudes	40.11 (10.35)

Prior to analyzing the mediation model, the total effect of CSA, CPA, and CN on APSA was estimated (i.e., path c). With no mediators in the model, the regression coefficient of APSA on CPA was statistically significant (path c; B=0.14, 95% CI=0.02–0.32, *p*<0.05), whereas the regression coefficient of APSA on CSA and CN was non-significant. Hence, the tested mediation model included only one of the three estimates of childhood maltreatment, that is, CPA.

**Fig. 1:**
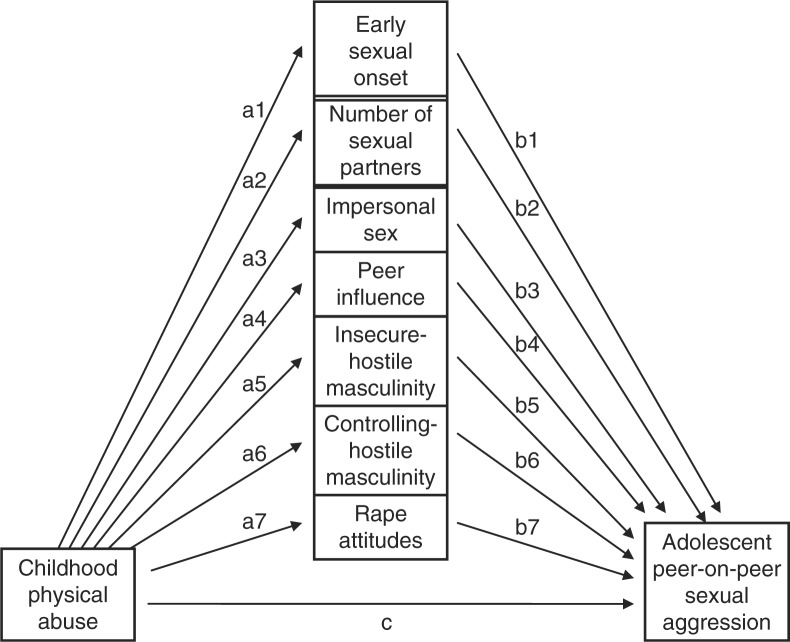
Childhood physical abuse, adolescent peer-on-peer sexual aggression, and mediators.

As seen in Fig. 1, the mediation model specified the effect of the independent variable (CPA) on the mediating variables, represented by early sexual onset (a1), number of sexual partners (a2), impersonal sex (a3), peer influence (a4), insecure-hostile masculinity (a5), controlling-hostile masculinity (a6), and rape attitudes (a7). The model also specified effects of the mediating variables on the dependent variable (APSA), represented by b1 to b7. Each of these effects was estimated along with the seven mediated effects (a1b1, a2b2, a3b3, a4b4, a5b5, a6b6, a7b7). In Fig. 1, path c represents the direct effect of CPA on APSA while controlling for the mediated effects.

The unstandardized significant estimates from the mediation model are reported in Table 2. The regression coefficients of the effects of CPA on six of the hypothesized mediators were positive and statistically significant (i.e., early sexual onset, number of sexual partners, peer influence, insecure-hostile masculinity, controlling-hostile masculinity, and rape attitudes), whereas only two of seven regression coefficients of the hypothesized effects of mediators on APSA were statistically significant (i.e., peer influence and insecure-hostile masculinity).

**Table 2:** Coefficients for the mediation model of childhood physical abuse and adolescent peer-on-peer sexual aggression

Mediator	Path a [95% CI]	Path b [95% CI]
Early sexual onset	0.21* [0.05–0.40]	*ns*
Number of sexual partner	1.31* [0.47–2.26]	*ns*
Impersonal sex	*ns*	*ns*
Peer influence	0.72* [0.26–1.30]	0.09* [0.03–0.13]
Insecure-hostile masculinity	3.96* [0.40–7.83]	0.01* [0.00–0.01]
Controlling-hostile masculinity	6.16* [0.76–11.17]	*ns*
Rape attitudes	6.30* [3.22–10.34]	*ns*

*Note*: CI=confidence interval.

* p<0.05.

The mediated effects of CPA on APSA via peer influence (path a4b4; B=0.06, 95% CI=0.01–0.16, *p*<0.05) and insecure-hostile masculinity (path a5b5; B=0.01, 95% CI=0.00–0.04, *p*<0.05) were significant, whereas the mediated effects of CPA on APSA via early sexual onset (path a1b1), number of sexual partners (path a2b2), impersonal sex (path a3b3), controlling-hostile masculinity (path a6b6), and rape attitudes (path a7b7) were non-significant. When the mediators were included in the model the path from CPA to APSA (path c) was not statistically significant.

## Discussion

The present study investigated potential pathways between three different forms of child maltreatment (sexual abuse, physical abuse, and neglect) and severe early APSA. Initial analyses indicated that CPA was significantly associated with APSA, whereas no significant link was found between measures of sexual abuse and neglect, and APSA. These findings correspond with Widom and Ames (1994) who documented that CPA was associated with later sexual aggression. Results are, however, at variance with the conclusions from a recent meta-analysis investigating adolescent sex offenders (Seto & Lalumiére, 2010), as the *sexually abused sexual abuser* hypothesis was not supported, indicating that a history of CSA did not increase risk of subsequent sexual aggressive behavior in the current study. Likewise, CN was not associated with APSA. Such inconsistencies may be explained because of variations in definitions and measurement, where statistical analysis for child maltreatment may be challenged by low prevalence rates, as was the case for reports on CSA in the present study. However, the prevalence rate for CN was compatible to the rate of CPA (7.9 and 8.2%, respectively), yet CN still did not predict APSA.

Interestingly, six out of seven mediators were significantly associated with physical abuse (early sexual onset, number of sexual partners, impersonal sex, controlling-hostile masculinity, and rape attitudes), whereas only two out of seven mediators were related to APSA (peer influence and insecure-hostile masculinity). Such findings suggest that the tested mediators were better linked with physical abuse than APSA. Even though the model indicated significant indirect effects, analyses could not determine, whether there were additional unmeasured mediators, whose variance were currently being ascribed to them.

According to the Hostile Masculinity path in the Confluence Model (Malamuth et al., 1991, 1995), men who have a history of family violence (comprising domestic violence, physical abuse, and/or sexual abuse) are more likely to develop adversarial or hostile schemata pertaining to intimate relationships. This contention was partly supported in the present study. The second Hostile Masculinity subscale, that taps into controlling-hostile masculinity, was only associated with CPA. In explaining such discrepancy, insecure-hostile masculinity may possibly better capture the developmental challenges of adolescence (Wolfe et al., 2004), as relationships between youth per se are characterized by insecurity about gender roles and sexual expectations (Lacasse & Mendelson, 2007). Although the Confluence Model is previously studied as a complex model comprising interaction of several components, the present study suggests that the contribution of separate elements of the model are indeed worth studying in relation to sexual aggression in non-criminal early adolescent populations.

Findings of the present study are further consistent with Wolfe and colleagues (Wolfe et al., 2004, 2006), who report that peer norms and pressure to behave in a certain manner function as important components of adolescent sexual aggression, because of motivation for compliance with peer groups. As such, the present study points to peer contexts as critical for implementation of rape prevention programs. Approaches such as peer-focused bystander interventions are one example where prevention messages are tailored not just to discourage perpetration, but also to highlight how peers contribute to the continuance of sexual aggression and the ways they can intervene to lessen perceived peer support (Banyard, Moynihan, & Plante, 2007). In addition, a recent study indicated that peer norms among male college students are crucial to men′s willingness to intervene against sexual aggression (Brown & Messman-Moore, 2009).

## Study limitations

A number of limitations from the present study need to be addressed. First, self-reports on adolescent APSA, as in other age cohorts of boys and men, may suffer from underreporting because of individual understanding and norms for sexually aggressive behaviors. Responses may be compromised in part due to lack of knowledge on what constitutes acceptable sexual behaviors (Lacasse & Mendelson, 2007). Respondents may not have perceived the coerciveness or force in their actions as others would. Also, behaviors that constitute rape under legal definitions may be regarded undesirable at the normative level, and thus deter respondents from disclosure (Maxwell et al., 2003). The present study used the multi-item measure of the SES, which is regarded as best practice, as it stimulates recall and disclosure through behaviorally-specific descriptions, rather than respondents’ comprehension of sexual assault or personal labels that they use to understand what they have done (Fisher, Cullen, & Daigle, 2005). However, despite the use of a multi-item measure, the present study may still suffer from potential underreporting of APSA. Second, child maltreatment was assessed retrospectively, using three single items that were taken from an interview, originally developed for adults. Consequently, the study failed to provide a clear operational definition of both abuse and neglect, which is possibly related to the low percentages obtained for CSA history. In addition, information on severity and frequency of maltreatment episodes was not included in the analyses. Even though poly-victimization has been shown to exist among children and youth, the present study did not address possible impacts of multiple trauma exposure, which may have influenced results. Third, because of the cross-sectional nature of the study, the direction of relationship between APSA and the proposed mediating variables could not be established. Additional research on the link between child maltreatment and later adolescent sexual aggression would benefit from a longitudinal approach for a better understanding of the underlying temporal sequencing. Fourth, the study was based on a relatively small sample of Danish adolescents, and concerns should be raised on the generalizability of study results, i.e., aspects of cultural norms on social and sexual practices specifically pertaining to Danish youth. Also, study findings are strictly applicable to early adolescents, and cannot be generalized to older age groups. Moreover, the measurement of impersonal sex may be methodologically flawed, which potentially influences results. Also, the impersonal sex items appear to measure normative behaviors for this age group of young boys, rather than impersonal sexual attitudes. Finally, the sample size may be too small to identify robust links between aversive childhood experiences and sexual aggression in adolescence. As such, study results indicate possible trends, which, however, need additional investigation to fully explain the developmental trajectories in question.

## Implications

With these limitations in mind, the present study adds to existing knowledge concerning the link between child maltreatment and adolescent sexual aggression, which has several implications for prevention programming. The study targets a group of non-criminal early adolescent boys with a history of child maltreatment, which, according to previous literature, is expected to be at a heightened risk of sexually aggressive behavior towards their female peers. Interrupting the link between child maltreatment and onset of juvenile sexual offending may be an important goal of prevention efforts (Seto & Lalumière, 2010).

Moreover, the study highlights two risk variables that appear to be subject to change: peer norms and insecure-hostile masculinity. By reinforcing the existing findings on the impact of peer norms on individual behavior, results support the importance of both special attention to boys at risk and also for prevention messages addressed to all boys, who even if they do not aggress themselves, can act in rape supportive ways. Rape prevention for boys may be more impactful that current efforts if it aims to involve the peer context and give all boys a role to play in reducing sexual aggression.

It is, however, imperative to acknowledge, that perpetration of sexual violence is the result of complex interplay between several factors, including developmental trajectories from child maltreatment to APSA (Barbaree & Marshall, 2008). The tested mediators are therefore to be placed within a larger context of factors driving the path from child maltreatment to early adolescent sexual violence perpetration.

## References

[CIT0001] Abbey A, McAuslan P, Zawacki T, Clinton A. M, Buck P. O (2001). Attitudinal, experimental, and situational predictors of sexual assault perpetration. Journal of Interpersonal Violence.

[CIT0002] Ageton S. S (1983). Sexual assault among adolescents.

[CIT0003] Banyard V. L, Moynihan M. M, Plante E. G (2007). Sexual violence prevention through bystander education: An experimental evaluation. Journal of Community Psychology.

[CIT0004] Barbaree H. E, Marshall W. L, Barbaree H. E, Marshall W. L (2008). An introduction to the juvenile sex offender. The juvenile sex offender.

[CIT0005] Black M. M, Oberland S. E, Lewis T, Knight E. D, Zolotor A. J, Litrownik A. J (2009). Sexual intercourse among adolescents maltreated before age 12: A prospective investigation. Pediatrics.

[CIT0006] Bollen K. A (1989). Structural equations with latent variables.

[CIT0007] Boyd N. J, Hagan M, Cho M. E (2000). Characteristics of adolescent sex offenders: A review of the research. Aggression and Violent Behavior.

[CIT0008] Bramsen R. H, Elklit A, Nielsen L. H (2009). A Danish model for treating victims of rape and sexual assault: The multidisciplinary public approach. Journal of Aggression, Maltreatment and Trauma.

[CIT0009] Bramsen R. H, Lasgaard M, Koss M. P, Shevlin M, Elklit A, Banner J (2013). Testing a multiple mediator model of the effect of childhood sexual abuse on adolescent sexual victimization. American Journal of Orthopsychiatry.

[CIT0010] Brown A. L, Messman-Moore T. L (2009). Personal and perceived peer attitudes supporting sexual aggression as predictors of male college students’ willingness to intervene against sexual aggression. Journal of Interpersonal Violence.

[CIT0011] Bunting B. P, Adamson G, Mulhall P (2002). A Monte Carlo examination of MTMM model with planned incomplete data structures. Structural Equation Modeling.

[CIT0012] Burton D. L, Miller D. L, Shill C. T (2002). A social learning theory comparison of the sexual victimization of adolescent sexual offenders and nonsexual offending male delinquents. Child Abuse and Neglect.

[CIT0013] Byers E. S, Eno R. J (1991). Predicting men's sexual coercion and aggression from attitudes, dating history, and sexual responses. Journal of Psychology and Human Sexuality.

[CIT0014] Casey E. A, Beadnell B, Lindhorst T. P (2009). Predictors of sexually coercive behavior in a nationally representative sample of adolescent males. Journal of Interpersonal Violence.

[CIT0015] de Bruijn P, Burrie I, van Wel F (2006). A risky boundary: Unwanted sexual behavior among youth. Journal of Sexual Aggression.

[CIT0016] DeKeseredy W. S, Kelly K (1995). Sexual abuse in Canadian university and college dating relationships: The contribution of male peer support. Journal of Family Violence.

[CIT0017] Efron B (1987). Better bootstrap confidence intervals. Journal of the American Statistical Association.

[CIT0018] Efron B, Tibshirani R. J (1993). An introduction to the bootstrap.

[CIT0019] Elklit A (2002a). Attitudes toward rape victims: An empirical study of the attitudes of Danish website visitors. Journal of Scandinavian Studies in Criminology and Crime Prevention.

[CIT0020] Elklit A (2002b). Victimization and PTSD in a Danish national youth probability sample. Journal of the American Academy of Child and Adolescent Psychiatry.

[CIT0021] Finkelhor D, Ormrod R, Turner H, Hamby S (2005). The victimization of children and youth: A comprehensive, National survey. Child Maltreatment.

[CIT0022] Finkelhor D, Ormrod R. K, Turner H (2007). Poly-victimization: A neglected component in child victimization. Child Abuse aand Neglect.

[CIT0023] Fisher B. S, Cullen F. T, Daigle L. E (2005). The discovery of acquaintance rape: The salience of methodological innovation and rigor. Journal of Interpersonal Violence.

[CIT0024] Foshee V. A, Benefield T. S, Ennett S. T, Bauman K. E, Suchindran C (2004). Longitudinal predictors of serious physical and sexual dating violence victimization during adolescence. Preventive Medicine.

[CIT0025] Gunby C, Woodhams J (2010). Sexually deviant juveniles: Comparisons between the offender and offence characteristics of child abusers and peer abusers. Psychology, Crime and Law.

[CIT0026] Jackson S. M, Cram F, Seymour F. W (2000). Violence and sexual coercion in high school students’ dating relationships. Journal of Family Violence.

[CIT0027] Johnson G. M, Knight R (2000). Developmental antecedents of sexual coercion in juvenile sexual offenders. Sexual Abuse.

[CIT0028] Kershner R (1996). Adolescent attitudes about rape. Adolescence.

[CIT0029] Kessler R. C, Sonnega A, Bromet E, Hughes M, Nelson C. B (1995). Posttraumatic stress disorder in the national comorbidity survey. Archives of General Psychiatry.

[CIT0030] Knight R. A, Sims-Knight J. E (2004). Testing an etiological model for male juvenile sexual offending against females. Journal of Child Sexual Abuse.

[CIT0031] Knight R. A, Sims-Knight J. E (2009). Using rapist risk factors to set an agenda for rape prevention.

[CIT0032] Koss M. P, Abbey A, Campbell R, Cook A, Norris J, Testa M (2007). Revising the SES: Collaborative process to improve assessment of sexual aggression and victimization. Psychology of Women Quarterly.

[CIT0033] Koss M. P, Oros C. J (1982). Sexual experience survey: A research instrument investigating sexual aggression and victimization. Journal of Consulting and Clinical Psychology.

[CIT0034] Lacasse A, Mendelson M. J (2007). Sexual coercion among adolescents. Journal of Interpersonal Violence.

[CIT0035] Lanier C. A (2001). Rape-accepting attitudes: Precursors to or consequences of forced sex. Violence Against Women.

[CIT0036] Lodico M. A, Gruber E, DiClemente R. J (1996). Childhood sexual abuse and coercive sex among school-based adolescents in a Midwestern state. Journal of Adolescent Health.

[CIT0037] Malamuth N. M, Linz D, Heavey C. L, Barnes G, Acker M (1995). Using the confluence model of sexual aggression to predict men's conflict with women: A 10-year follow-up study. Journal of Personality and Social Psychology.

[CIT0038] Malamuth N. M, Sockloskie R. J, Koss M. P, Tanaka J. S (1991). Characteristics of aggressors against women: Testing a model using a national sample of college students. Journal of Consulting and Clinical Psychology.

[CIT0039] Maxwell C. D, Robinson A. L, Post L. A (2003). The nature and predictors of sexual victimization and offending among adolescents. Journal of Youth and Adolescence.

[CIT0040] Muthén L. K, Muthén B. O (1998–2009). Mplus: Version 5.21.

[CIT0041] Preacher K. J, Hayes A. F (2008). Asymptotic and resampling strategies for assessing and comparing indirect effects in multiple mediator models. Behavior Research Methods.

[CIT0042] Raj A, Silverman J. G, Amaro H (2000). The relationship between sexual abuse and sexual risk among high school students: Findings from the 1997 Massachusetts youth risk behavior survey. Maternal and Child Health Journal.

[CIT0043] Seto M. C, Kjellgren C, Priebe G, Mossige S, Svedin C. G, Langström N (2010). Sexual coercion experience and sexual coercive behavior: A population study of Swedish and Norwegian male youth. Child Maltreatment.

[CIT0044] Seto M. C, Lalumière M. L (2010). What is so special about male adolescent sexual offending? A review and test of explanations through meta-analysis. Psychological Bulletin.

[CIT0045] Thomas T. A, Fremouw W (2009). Moderating variables of the sexual “victim to offender” cycle in males. Aggression and Violent Behavior.

[CIT0046] Vega V, Malamuth N. M (2007). Predicting sexual aggression: The role of pornography in the context of general and specific risk factors. Aggressive Behavior.

[CIT0047] Ward C (1988). The attitudes toward rape victims scale: Construction, validation, and cross-cultural applicability. Psychology of Women Quarterly.

[CIT0048] White J. W, Kadlec K. M, Sechrist S, Barbaree H. E, Marshall W. L (2008). Adolescent sexual aggression within heterosexual relationships. The juvenile sex offender.

[CIT0049] White J. W, Smith P. H (2004). Sexual assault perpetration and reperpetration: From adolescence to young adulthood. Criminal Justice and Behavior.

[CIT0050] Widom C. S, Ames M. A (1994). Criminal consequences of childhood sexual victimization. Child Abuse and Neglect.

[CIT0051] Wolfe D. A, Jaffe P. G, Crooks C. V (2006). Adolescent risk behaviors: Why teens experiment and strategies to keep them safe.

[CIT0052] Wolfe D. A, Wekerle C, Scott K, Straatman A, Grasley C (2004). Predicting abuse in adolescent dating relationships over 1 year: The role of child maltreatment and trauma. Journal of Abnormal Psychology.

[CIT0053] Xenos S, Smith D (2001). Perceptions of rape and sexual assault among Australian adolescents and young adults. Journal of Interpersonal Violence.

[CIT0054] Young A. M, King L, Abbey A, Boyd C. J (2009). Adolescent peer-on-peer sexual aggression: Characteristics of aggressors of alcohol and non-alcohol-related assault. Journal of Studies on Alcohol and Drugs.

